# Acute fibrinous and organizing pneumonia: two case reports and literature review

**DOI:** 10.1186/s12890-019-0861-3

**Published:** 2019-08-05

**Authors:** Jingjing Lu, Qi Yin, Yunlan Zha, Shuangshuang Deng, Jianhao Huang, Zhongliang Guo, Qiang Li

**Affiliations:** 1 Department of Respiratory Medicine, Shanghai East Hospital, Tongji University School of Medicine, Pudong, 200120 Shanghai China; 2 Department of Pathology, Shanghai East Hospital, Tongji University School of Medicine, Pudong, 200120 Shanghai China

**Keywords:** Acute fibrinous and organizing pneumonia

## Abstract

**Background:**

Acute fibrinous and organizing pneumonia (AFOP) is a very rare form of acute or subacute lung injury, which is characterized by patches of “fibrin balls” distributed within the alveoli.

Given the lack of typical clinical manifestations, AFOP is often misdiagnosed as pneumonia, tuberculosis, etc. Definitive diagnosis is obtained from a lung biopsy. Corticosteroids are usually effective.

**Case presentation:**

We report two cases of patients with histopathological manifestations of AFOP, which were significantly improved after corticosteroid therapy. Previous reports of the clinical and pathological characteristics of AFOP were reviewed to improve clinicians’ understanding of this disease.

**Conclusions:**

Early identification and diagnosis are very important for AFOP treatment. The prognosis is acceptable after timely and effective treatment.

## Background

Acute fibrinous and organizing pneumonia (AFOP) is a very rare form of acute or subacute lung injury, characterized by patches of “fibrin balls” distributed within the alveoli. This unique histological feature distinguishes AFOP from other more common histological patterns such as diffuse alveolar damage (DAD), which has classic fibrinous hyaline membranes lining the alveoli. AFOP can either be idiopathic or concomitantly occur with other lung diseases such as infections, collagen vascular diseases, adverse drug or chemical reactions, hematological malignancy, altered immune status, and inhalation diseases. Definitive diagnosis is obtained from a lung biopsy due to the lack of other specific detection methodologies. Corticosteroids are commonly used to treat AFOP, although no standard regimen has been established.

## Case presentation

### Case 1

A 70-year-old man was admitted with a cough and fever since two days. His blood pressure was 120/74 mmHg, heart rate was 72 beats/min, respiratory rate was 20 breaths/min, temperature was 38.2 °C and oxygen saturation was 98% on oxygen supplementation with a nasal cannula that delivered oxygen at a rate of 2 L/min.

Initial laboratory data included a white blood cell count (WBC) of 7.41*10^9/L, hemoglobin (HB) 132.0 g/L, platelets (PLT) 387.0*10^9/L, red blood cell count (RBC) of 4.27*10^12/L, ESR 72 mm/H, CRP > 150 mg/L, IL-6 88.19 pg/ml, PCT 0.244 ng/ml. Arterial blood, hepatorenal function, electrolytes, respiratory virus test, fungi test were normal. The blood cultures revealed no growth, while sputum grew the normal respiratory tract flora. Three consecutive samples for acid-fast bacilli were also negative. There was no other identifiable source of infection in the body. Autoimmune work-up including anti-neutrophil antibody, anti-neutrophil cytoplasmic antibody, and rheumatoid factor were all negative.

**Fig. 1:**
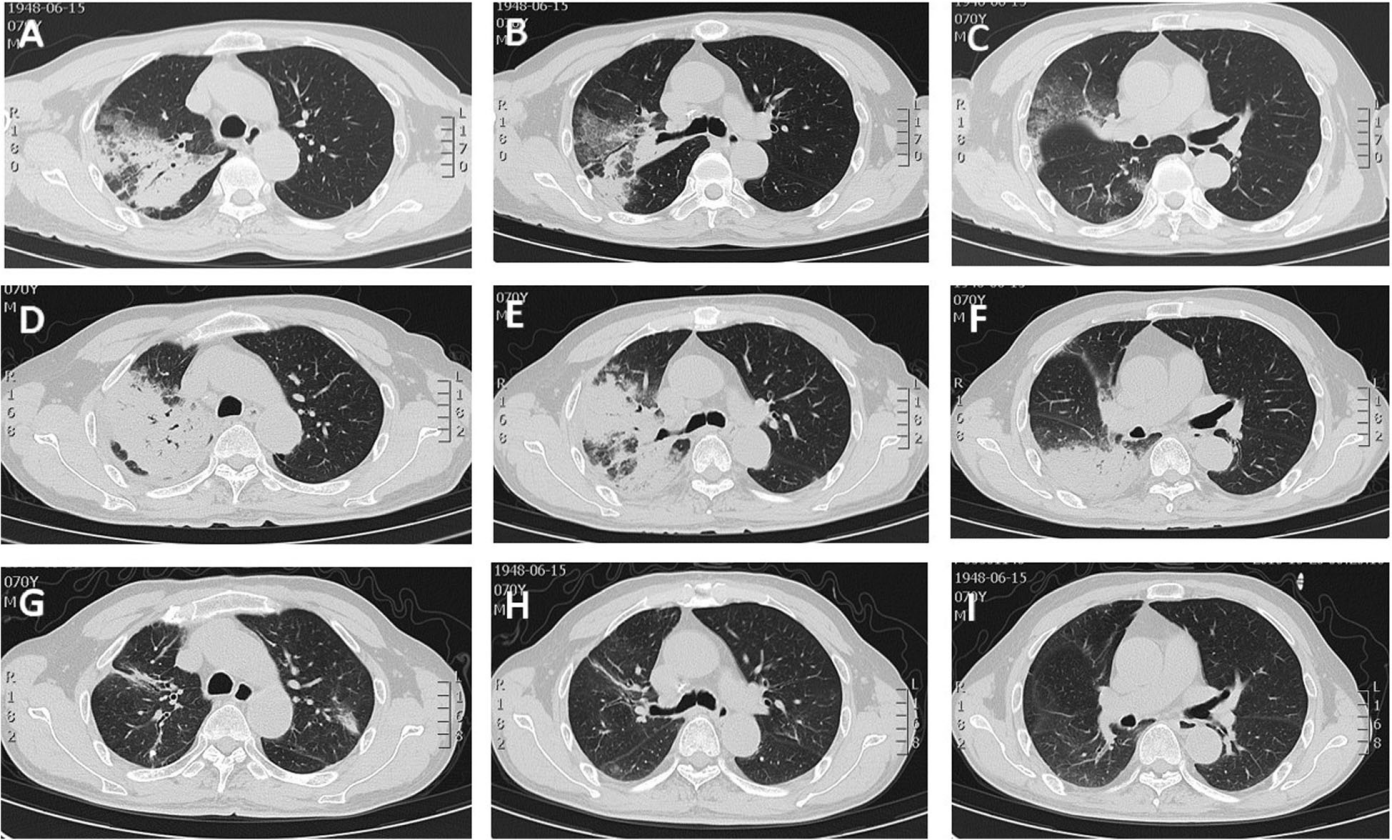
In case 1, CT scan (**a**, **b** and **c**) showed consolidation of the right upper lobe with an air bronchogram. Ten days after treatment with antibiotics, repeat CT scan (**d**, **e** and **f**) showed more extensive lesion than previously observed. After treatment with corticosteroids, CT scan (**g**, **h** and **i**) showed that the lesion was absorbed

No obvious abnormalities were found in ECG, abdominal ultrasound, thyroid ultrasound, and lower limb vascular ultrasound. A computed tomography (CT) scan of the chest (Fig. 1a, b and c) showed right upper lobe consolidation, patchy infiltrates, and a “ground-glass” appearance in the right lower lobes. The patient was administered empirical antibiotic therapy for community-acquired pneumonia, which did not improve his condition. The second CT scan (Fig. 1d, e and f) performed 10 days after antibiotic treatment showed that the lesion was denser and more extensive than before.

**Fig. 2:**
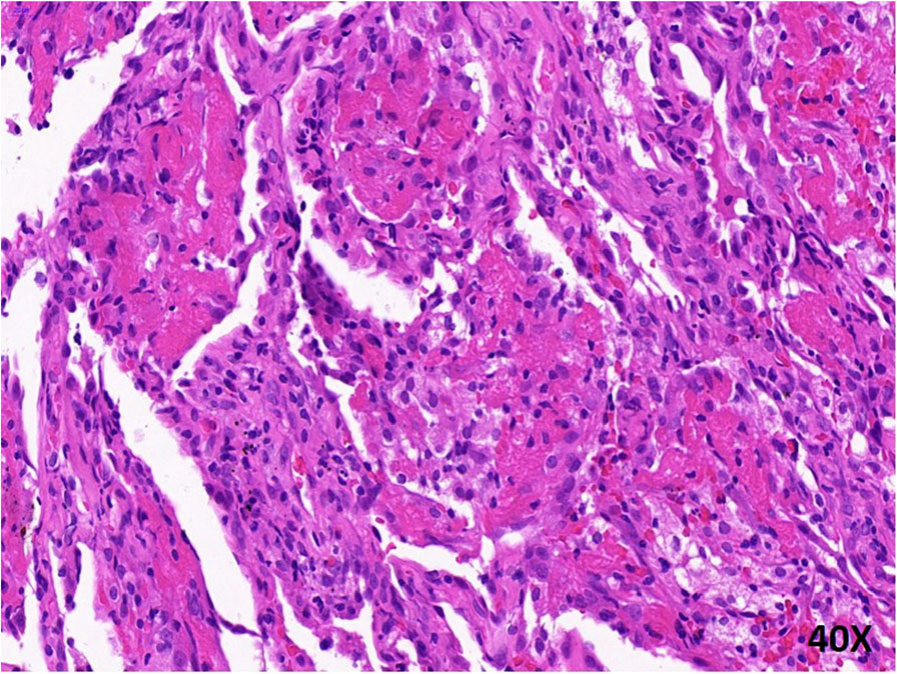
Histopathology of the lung showed multiple organizing “fibrin balls” within alveolar spaces, without associated hyaline membrane formation at 400X

Endobronchial ultrasonography with a guide sheath (EBUS-GS) was performed to retrieve about eight pieces of tissue from B^2a^ in upper lobe of the right lung. Histological examination revealed prominent fibrinous exudation within most of the alveolar spaces with “fibrin balls” formation (Fig. 2). The next generation sequencing (NGS) was performed with lung tissues and bronchoalveolar lavage fluid (BALF), which did not detect any bacteria, fungi, DNA viruses and parasites.

A diagnosis of AFOP was made. Therefore, the patient was started on methylprednisolone 40 mg intravenous drip twice a day. His temperature was back to normal and his symptom of cough lessened. The patient was discharged on a tapering schedule of methylprednisolone 24 mg once a day. After 1.5 months, a CT scan (Fig. 1g, h and i) showed that the bilateral lung lesions had absorbed more clearly than before, leaving only a few fibrous strands. Then, we adjusted his dose to 20 mg, and the patient is in stable condition with outpatient follow-up.

### Case 2

A 71-year-old woman had a history of controlled type 2 diabetes mellitus. She received traditional Chinese acupuncture treatment for lower limb pain two months ago, and had local swelling for one week. She recently had an upper respiratory infection and visited our emergency department when her shortness of breath worsened, fever persisted and sputum thickened for a week. She had no history of smoking, respiratory, heart, liver or kidney diseases.

Her pulse rate was 98/min, blood pressure 128/80 mmHg, temperature was 38.9 °C and respiratory rate was 22/min. Her oxygen saturation was 90% on room air. Auscultation revealed crackles in the lower lung fields and dullness to percussion at the bases.

The arterial blood gas showed that the pH value was 7.40, the partial pressure of oxygen was 70 mmHg, the partial pressure of carbon dioxide was 40 mmHg. The WBC count was 8.71*10^9/L (differentials 74% neutrophils), ESR 66 mm/H and CRP 102 mg/L. Blood culture showed no growth, and sputum growth showed normal respiratory flora. There were no other identifiable sources of infection in the body. Autoimmune tests including anti-neutrophil antibody (ANA), anti-neutrophil cytoplasmic antibody (ANCA) and rheumatoid factor (RF) were negative.

**Fig. 3:**
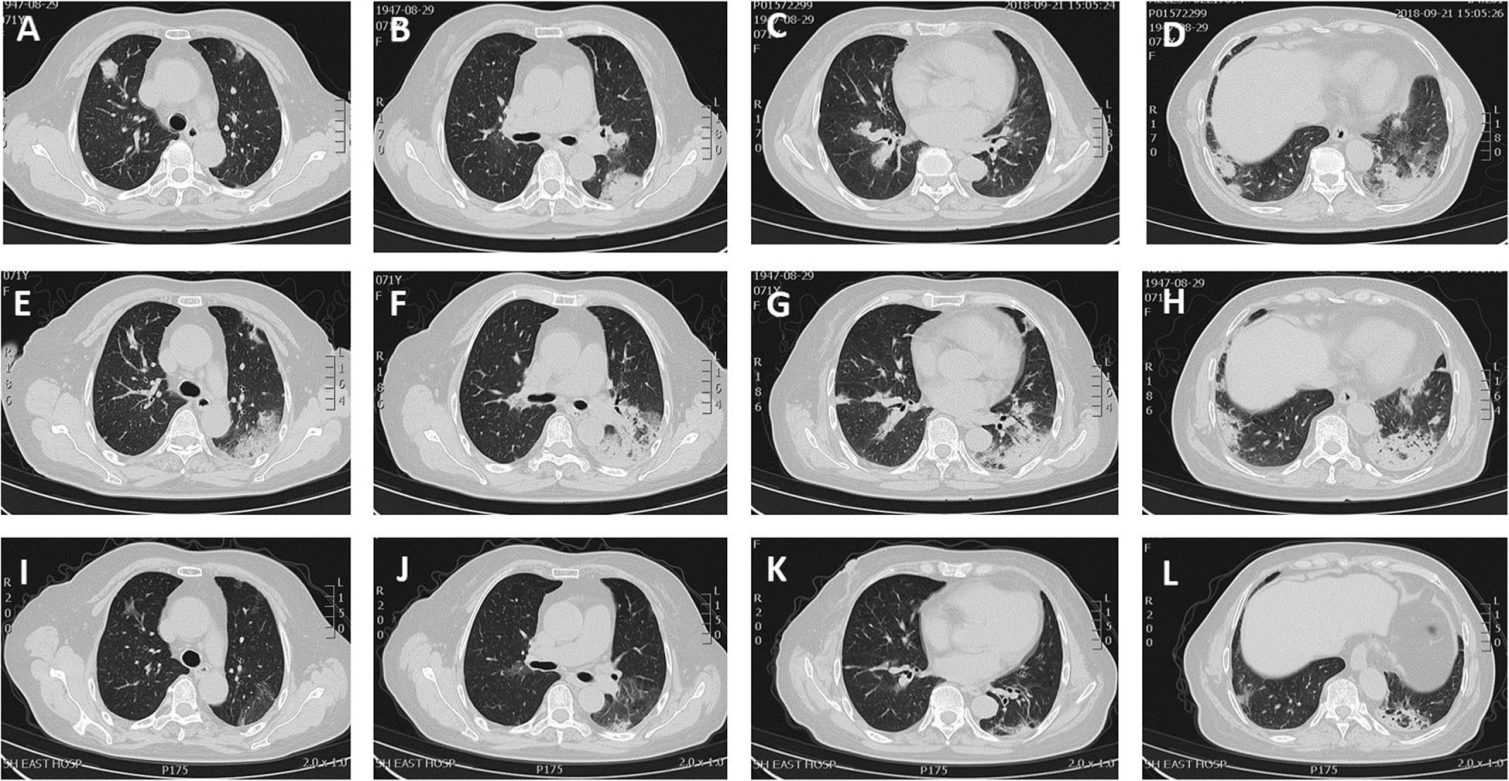
In case 2, CT scan (**a**, **b**, **c** and **d**) showed geographic and patchy areas of ground glass opacities with interlobular septal thickening, predominantly in basal portions of the lungs. Repeat CT scan (**e**, **f**, **g** and **h**) after treatment with antibiotics showed diffuse bilateral ground glass opacities. CT images (**i**, **j**, **k** and **l**) obtained 1.5 months after the initial study demonstrated clearing of diffuse opacities, with residual peribronchial fibrosis and distortion

The CT scan showed (Fig. 3a, b, c and d) bilateral ground glass opacification (GGO) and haze, with more prominence in the upper lobe of the left lung. Initial diagnosis was community-acquired pneumonia and the patient was treated with moxifloxacin 400 mg intravenously once daily plus piperacillin-tazobactam 4.5 g twice a day. However, the symptoms improved minimally. CT scan (Fig. 3, f, g and h) was performed 2 weeks after antibiotic therapy, which showed worsening of the lung lesions.

**Fig. 4:**
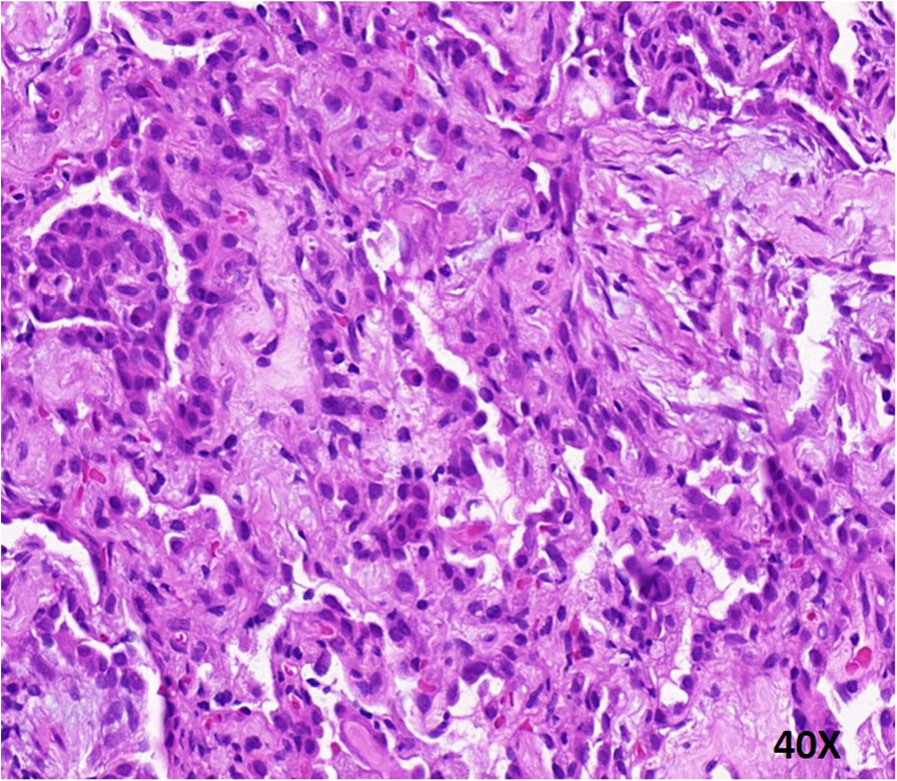
Photomicrograph revealed “fibrin balls” in the airspaces alternating with organizing fibroblastic tissue at 400X

Bronchoscopy and EBUS-GS-guided biopsy were performed. We obtained about 10 small tissues from B^1 + 2b^ and B^1 + 2c^ in upper lobe of the right lung. Histological examination revealed multiple organizing “fibrin balls” within alveolar spaces, without associated hyaline membrane formation. (Fig. 4). Staining for fungus, acid-fast bacilli and Gram staining were all negative. The lung tissues were examined by the NGS technology, which showed no bacteria, fungi, DNA viruses and parasites.

After the final diagnosis of AFOP, we started intravenous injection of prednisolone 1 mg/kg daily. The patient was clinically better and discharged after 10 days, and recommended to take oral prednisolone 40 mg.

The CT scan (Fig. 3i, j, k and l) showed interval improvement after 1.5 months of treatment with 40 mg oral prednisolone daily. Currently, she is on a tapering dose of steroids and completely symptom-free.

## Discussion and conclusions

AFOP is a type of acute or subacute lung injury characterized by a dominant histological pattern of patchy intra-alveolar fibrin and organizing pneumonia, which does not meet the histological criteria for patterns of diffuse alveolar damage (DAD), organizing pneumonia (OP), or eosinophilic pneumonia (EP). The first report in 2002 by Beasley [1] included 17 male patients, with the average age of 62 years (range, 50–70 years).

AFOP was histologically distinguished from DAD, bronchiolitis obliterans with organizing pneumonia (BOOP) and EP in the setting of acute lung injury.

The etiology of AFOP is unclear and may be associated with multiple factors, such as autoimmune diseases (ankylosing spondylitis, anti-phospholipid syndrome, anti-synthetase syndrome, dermatomyositis, systemic lupus erythematosus), drugs (abacavir, amiodarone, bleomycin, decitabine, everolimus, sirolimus, zacytidine), environmental causes (aerosols, asbestos, coal, dusts), infections (Acinetobacter baumannii, Aspergillus fumigatus, Chlamydia pneumoniae, Cytomegalovirus, H1N1 influenza, *Haemophilus influenzae*, histoplasmosis, human immunodeficiency virus, *Pneumocystis jirovecii*) and transplantation (allogenic hematopoietic stem cell transplant, lung transplant) [2, 3]. The two patients in this case report did not have possible related infections, drugs, environmental exposure or chemical exposure. Many idiopathic cases of AFOP have been previously reported [4–7].

AFOP can have various symptoms and signs. Clinical manifestations of AFOP appear to lack specificity. The most common symptom is a dry cough. Other symptoms include shortness of breath, hemoptysis, chest pain, fever, fatigue, anorexia, loss of weight and night sweat [8]. Fever is another predominant manifestation and the highest temperature can reach more than 39 °C. Laboratory tests may show elevated inflammatory cytokines, which is nonspecific.

The two patients presented symptoms of community-acquired pneumonia. Inflammatory markers, such as CRP, WBC and IL-6 were elevated. The symptoms did not obviously improve after regular anti-infective treatment, and the pulmonary lesions were more serious than before. This prompted us to consider bronchoscopy to obtain a tissue diagnosis since there are many similarities in clinical manifestations, laboratory examination, imaging characteristics, etc. Based on the clinical course of our patients, AFOP should be considered in the differential diagnosis in an individual unresponsive to standard antibiotic therapy with poorly progressing pneumonia.

The role of important diagnostic modalities for obtaining diagnosis need to be discussed. HRCT provides valuable inputs from the lesion site. It also helps to determine the effectiveness of anti-infection therapy. Many different radioactive manifestations of AFOP have been described [8]. Numerous images of AFOP showed involvement of both lungs, such as GGO, consolidation, nodular shadows, interlobular septal thickening, etc. Single occupying mass was also reported [9]. Some patients have unilateral nodular consolidation shadow, which is easily confused with tuberculosis, tumor, etc. In our patients, the bilateral lung fields showed scattered GGO, diffuse infiltration, partial consolidation and bronchial inflammation in CT scan. Similarities with common pneumonia is also one of the reasons for misdiagnosis.

Pathological diagnosis is very important for AFOP. In the two cases, we obtained tissue samples through EBUS-GS-guided biopsy. EBUS-GS is a novel method used for collecting peripheral pulmonary lesion samples. It is performed by introducing a guide sheath-covered miniprobe into the target bronchus and withdrawing the miniprobe after lesion detection, leaving the guide sheath in situ as a working channel for obtaining lesion samples. Lesion samples can then be obtained through the guide sheath with a brush, biopsy forceps, or other devices [10]. Ikezawa [11] reported successfully diagnosing 38 out of 67 lesions (58%) by EBUS-GS under X-ray fluoroscopy, including five pure GGO lesions. Another study included 40 GGO lesions and the overall diagnostic yield of EBUS-GS-guided transbronchial biopsy was 65% [12]. In addition, the diagnostic yield was 68.4% for GGO ≥20 mm and 61.9% for GGO < 20 mm (*p* > 0.05). Additionally, video-assisted thoracic surgery biopsy (VATS) and CT-guided biopsy were commonly used to obtain samples. As compared to VATS and CT-guided biopsy, EBUS-GS-guided biopsy is less invasive and simpler.

Sampling limitation may lead to misdiagnosis because small biopsy tissues might not represent the intrinsic lesions. Feng [13] reported two cases of lung consolidation and occupying lesions with typical histological presentation of AFOP, but the final diagnosis was tuberculosis and tumor. They considered that even though the microscopic findings were very typical, it could be a reactive change in the peripheral tissue reacting to the intrinsic lesion. In such cases, sufficient samples are critical. The application of EBUS-GS can help the bronchoscope to squeeze into the softer distal bronchus, which is closer to the peripheral lesions, so as to reduce the possibility of straggles into other airways when the biopsy forceps are re-inserted, which is more conducive to improving the diagnostic rate. Dynamic monitoring of the miniprobe ensures that part of the biopsy is located in the center of the lesion rather than at the edge. The number of biopsies and the positive rate are closely related to the operator’s technique. In our patients, the lung tissue samples obtained by bronchoscopy biopsy were sufficient. The pathology confirmed the characteristics of AFOP, and the lesions were absorbed after treatment. So the diagnosis of AFOP was reliable.

The next generation sequencing (NGS) technology, characterized by high accuracy and high throughput, is playing an increasingly important role in clinical practice. In our two patients, lung tissues and alveolar lavage fluid were both examined by NGS, and no definite etiological evidence was found, which further confirmed the final diagnosis of AFOP. We believe that NGS technology can detect more pathogens due to its high throughput and sensitivity. It has a very broad prospect not only in identification of pathogens in infectious diseases but also in differential diagnosis of infectious and non-infectious diseases.

AFOP can be treated by antibiotics, corticosteroids, immunosuppressants (mycophenolate mofetil, azathioprine, and cyclophosphamide), etc. Since there is no ideal treatment for AFOP, the treatment of choice and therapy duration should be decided based on the medical course and etiology, with some patients being dependent on corticosteroids, immunosuppressants, and oxygen [14]. At present, the main therapeutic drug of AFOP is corticosteroids, but there are no standard treatment guidelines for dose and course [15]. Prednisolone shock therapy with sequential oral administration of prednisolone tablets is generally recommended. This treatment was also used for our patients, which achieved good results. The course of treatment should be determined according to the clinical manifestations, blood gas and imaging changes of patients [16]. Long-term corticosteroid therapy is associated with better outcomes.

Two variants of the disease have been described. An acute variant usually manifests as rapid respiratory failure, which always needs mechanical ventilation, and the mortality rate is nearly 100%. The other variant, which is subacute, usually does not progress to respiratory failure. It often needs long-term corticosteroid therapy, and has a good prognosis. Both our patients were classified as subacute, which did not cause severe respiratory failure and had favorable prognosis. Histological changes in patients with AFOP should be circumspectly treated and closely monitored. The two patients in this report are continuing to take their medicine regularly and following-up on time. Another biopsy will be required if and when the clinical conditions progress or worsen.

## Abbreviations

AFOP: Acute fibrinous and organizing pneumonia;
ANA: Autoimmune work up including anti neutrophil antibody
ANCA: Anti nuetrophil cytoplasmic antibody
BALF: Bronchoalveolar lavage fluid
BOOP: Bronchiolitis obliterans with organizing pneumonia
CRP: C-reactive protein
CT: Computed tomography
DAD: Diffuse alveolar damage
EBUS-GS: Endobronchial ultrasonography with a guide sheath
EP: Eosinophilic pneumonia
ESR: Erythrocyte sedimentation rate
HB: Hemoglobin
IL-6: Interleukin-6
NGS: The next generation sequencing
OP: Organizing pneumonia
PLT: Platelets
RBC: Blood cell count
RF: Rheumatoid factor
VATS: Video-assisted thoracic surgery
WBC: White blood cell count
